# Creatine: Clinical Implications for Orthopedic Surgeons

**DOI:** 10.7759/cureus.103937

**Published:** 2026-02-19

**Authors:** Samer Al-Humadi, Kevin Kohut, Patrick J Denard

**Affiliations:** 1 Orthopedic Surgery, Kaleida Health Olean General Hospital, Olean, USA; 2 Orthopedic Surgery, Orthopedic Center of Illinois, Springfield, USA; 3 Orthopedic Surgery, Oregon Shoulder Institute, Medford, USA

**Keywords:** athletes, creatine, exercise, nutritional supplement, phosphocreatine, safety, sarcopenia

## Abstract

Many athletes and active individuals use nutritional supplements for performance, muscle mass, and recovery. Creatine developed popularity in the 1990s and has continued today. During high-intensity exercise, anaerobic glycolysis and the phosphocreatine shuttle provide adenosine triphosphate (ATP). In older adults, creatine supplementation with resistance training has been shown to increase muscle mass and strength in some studies. Evidence to support creatine supplementation in adolescent and pediatric patients is limited. The most common side effect is weight gain, and there do not appear to be any adverse effects on renal function in healthy athletes. In the few studies to date, the role of creatine in postoperative recovery has not been clearly established. This review summarizes the basic science of creatine, its potential role in recovery and supplementation, and its safety profile.

## Introduction and background

**Figure 1:**
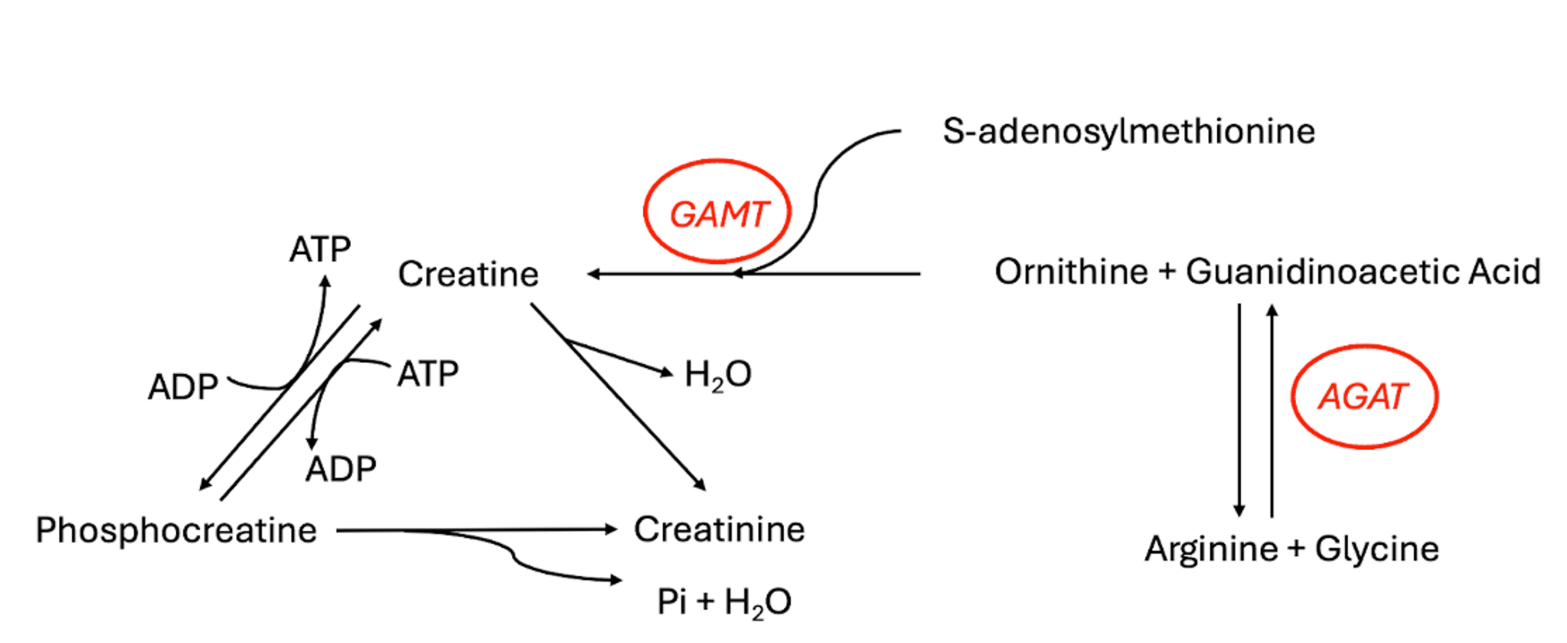
Creatine synthesis GAMT: Guanidinoacetate N-methyltransferase; AGAT: Arginine:glycine amidinotransferase. Image credits: Adapted with permission from Kreider and Jung [7].

Owing to the popularity of nutritional supplements, the orthopedic surgeon will surely encounter patients taking creatine. It is a naturally occurring nitrogenous organic amino acid compound acquired by reactions involving amino acids arginine, methionine, and glycine [1]. Creatine is naturally found in red meat and seafood, with trace amounts found in some plants [1-3]. Depending on muscle mass, humans require 1-3 g of creatine per day to maintain adequate stores [4]. As an example, a 70 kg male requires about 2 g of creatine per day, half of which can be obtained through the diet [5]. This includes but is not limited to salmon (4.5 g/kg of creatine), tuna (4 g/kg), herring (6.5-10 g/kg), beef (4.5 g/kg), pork (5 g/kg), and milk (0.1 g/kg) [6]. Aside from dietary sources, creatine can be endogenously synthesized in the liver, kidneys, and pancreas. About 95% of creatine is found within skeletal muscle, with the remaining 5% in the brain, testes, and heart. Of intramuscular creatine, about one-third is free creatine, and two-thirds is phosphocreatine [3,6]. In the liver and kidneys, glycine and arginine are processed by the enzyme arginine:glycine amidinotransferase (AGAT) and subsequently methylated to produce creatine (Figure 1) [4,7]. Approximately 1%-2% of intramuscular creatine is degraded into creatinine and excreted in urine [3]. For a 70 kg male with an approximate creatine pool of 120 g, this is about 2 g per day.

Creatine gained popularity in the 1990s and has continued to be a frequently consumed supplement for performance and recovery. The 2013 National Collegiate Athletic Association (NCAA) National Study of Substance Use Habits of College Student Athletes found that 13.2% used creatine in the last year [8]. Beyond athletes and active individuals, creatine supplementation has been investigated as a possible supplement for elderly individuals. The US Anti-Doping Agency (USADA), the International Olympic Committee, and the NCAA do not prohibit creatine use [9]. However, they are not regulated by the US Food and Drug Administration, and thus products may not contain precise or intended ingredients. Providers should educate patients on this and encourage them to research a potential supplement’s ingredients and source. The purpose of this review is to educate orthopedic surgeons on creatine basic science, nutrition, roles in patient function and recovery, and safety.

## Review

Mechanism of action

**Figure 2:**
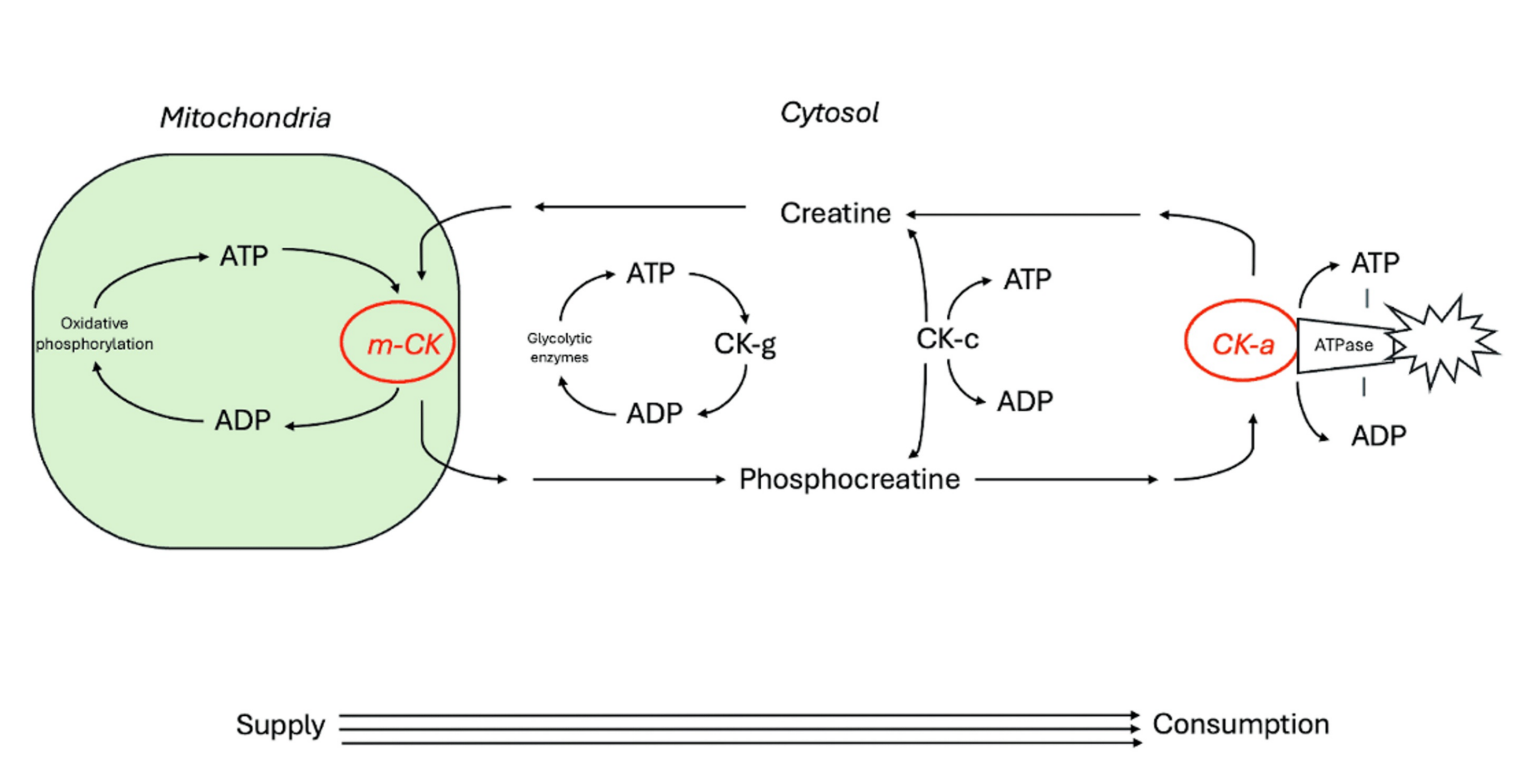
Creatine and phosphocreatine system for energy buffering m-CK: Mitochondrial creatine kinase; CK-g: Creatine kinase coupled to glycolytic enzymes; CK-c: Cytosolic creatine kinase. Image credits: Adapted with permission from Wallimann et al. [10].

The main function of creatine is in the creatine kinase pathway with temporal and spatial energy buffering [10]. Dietary and endogenous creatine are transported into target cells via a creatine transporter. Within the cell, microcompartments in the cytosol and mitochondria work to maintain an equilibrium of creatine/phosphocreatine and adenosine triphosphate/adenosine diphosphate (ATP/ADP) [10]. The contribution of these microcompartments depends upon cell type. The creatine kinase system stabilizes cellular ATP levels, maintaining a high ATP/ADP ratio and enabling efficient ATP use. While some creatine kinase processes are strictly ATP-consuming (such as myofibrillar acto-myosin ATPase, sarcoplasmic reticulum calcium-ATPase, plasma membrane sodium/potassium-ATPase, and ATP-gated potassium channels), creatine kinase regenerates ATP from phosphokinase. The system provides a temporal buffer by building up large cytosolic stores of phosphocreatine. In the heart, this is predominantly from oxidative phosphorylation. Within skeletal muscle, this is from glycolysis and oxidative phosphorylation. Creatine is transported into the cell and changed to phosphocreatine by cytosolic creatine kinase, creatine kinase coupled to glycolysis, or by mitochondrial creatine kinase coupled to oxidative phosphorylation (Figure 2) [10]. Mitochondrial creatine kinase, located in the intermembrane space, uses mitochondrial ATP to produce phosphocreatine. This use of ATP is most important for refilling phosphocreatine energy stores in oxidative tissues, such as for extensive muscle contraction. With short, high-intensity exercise, muscle ATP is obtained from anaerobic glycolysis and phosphocreatine [11]. The phosphocreatine shuttle is the main ATP source during maximum effort exercise under 10 seconds [11,12]. Thus, increasing phosphocreatine stores is thought to improve muscle endurance and performance [12]. Creatine supplements downregulate endogenous production; however, this returns to baseline once supplementation is stopped [12].

Dosing

Orthopedic surgeons may be asked about dosing and loading by patients in the clinic setting, as loading doses for creatine prior to a daily maintenance dose have been considered and investigated. One loading method involves taking 5 g creatine monohydrate (or about 0.3 g/kg body weight) four times a day for five to seven days to saturate muscle creatine stores followed by maintenance of 3-5 g per day [4]. A crossover, double-blind, randomized controlled trial investigated creatine loading on maximal strength and endurance [13]. Subjects received either placebo or 20 g creatine monohydrate per day for five days. The creatine group had significantly increased number of repetitions (14.7% increase; p = 0.036) and total work (11.1% increase; p = 0.038). However, the study was limited by small sample size and only male participants. Furthermore, supplementation had no effect on maximum strength, rating perceived exertion (RPE), or fatigue index. Hultman et al. examined loading versus gradual supplementation strategies [3]. Loading with 20 g/day for six days followed by 2 g/day for 30 days was compared to daily gradual supplementation with 3 g/day over 28 days. The authors found that creatine muscle accumulation was similar in both groups with a 20% increase; however, this study was also limited by low sample size of 31 participants. While it does not appear necessary for athletes to load, those that wish to maximize physical performance in a short period (less than 30 days) may consider a creatine loading strategy, whereas those who plan to supplement creatine over an extended period (more than 30 days) may be able to use a maintenance dose alone [14].

Safety

When creatine initially became popular, safety concerns were raised. However, more recent data has suggested that the risk of supplementation is low. Early case studies reported kidney dysfunction in individuals taking creatine with or without other supplements. One study on rats with cystic renal disease found that feeding creatine exacerbated kidney disease [4]. Case studies have reported individuals presenting with acute renal failure after creatine supplementation. However, it is difficult to draw conclusions from these studies as confounding variables limit their value [15]. In contrast, other studies have found no adverse renal effects in healthy individuals and athletes. A recent umbrella review of systematic reviews and meta-analyses found minimal side effects with minimal data to support renal damage [16]. Kreider et al. found no significant effects on renal function in Division IA college football players taking creatine supplements for 21 months [17]. This study was limited by lack of randomization and sample size, as only 17 players took creatine for 12-21 months, while 35 athletes took creatine for only 0-12 months.Another systematic review and meta-analysis investigated possible renal function side effects [18]. The study found no significant changes to serum creatinine and plasma urea levels.

The most consistently reported side effect has been weight gain [4]. Early water retention in the first few days has been described. One randomized controlled trial investigating the effects of three days of creatine supplementation found an increase in body water and extracellular body water [19]. However, this increase in body water does not appear to remain long-term. Other studies have shown no increase in total body water after long-term use. One study showed no increase in body water (p = 0.99), intracellular fluid (p = 0.44), or extracellular body fluid (p = 0.69) after five weeks of creatine supplementation [20]. Finally, a study on creatine supplementation in adult and pediatric patients with neurodegenerative conditions have shown that it is well tolerated with long-term use (up to 30 g/day for five years) [4]. Overall, these studies suggest creatine is safe with minimal effects on renal function in healthy individuals. However, it has been recommended to avoid supplementation in those with poor kidney function [15].

Diet and creatine timing

It is important for the orthopedic surgeon to be familiar with patient dietary restrictions. Athletes may have certain food restrictions stemming from perceived health benefits, ethical reasoning, and religious or philosophical beliefs. Lacto-ovo-vegetarians do not eat animals but will consume eggs and dairy products. Lacto-vegetarians consume plant-based food and dairy. Ovo-vegetarians consume plant-based food and eggs. Vegans do not eat any animal products. These diets decrease muscle creatine stores as food sources rich in creatine are excluded [21,22]. Burke et al. compared resistance training with creatine supplementation in vegetarians and non-vegetarians using vastus lateralis muscle biopsies to assess change in muscle and body composition, fluid status, and muscle creatine [22]. Vegetarian baseline biopsy samples (117 mmol.kg^-1^) showed significantly lower total creatine (sum of free creatine and phosphocreatine) at baseline compared to non-vegetarians (130 mmol.kg^-1^; p < 0.05). Interestingly, vegetarians were found to have an even greater increase in lean tissue mass (2.4 kg versus 1.9 kg in non-vegetarians; p < 0.05), type-II muscle fiber area (p < 0.05), muscle total creatine (p < 0.05), and muscle performance with one rep-max (p < 0.05) compared with non-vegetarians. Thus, supplements in this patient population may be beneficial.

Timing of creatine consumption has become an area of interest. Mechanisms for the effects of creatine timing in addition to resistance training are currently not completely understood [23]. Hypothetical mechanisms include exercise-induced hyperemia allowing for greater creatine delivery to muscle. Currently, pre-exercise and post-exercise creatine ingestion appear to produce similar results. One double-blind randomized controlled trial was performed on 23 professional cyclists to investigate high-dose, short-term creatine supplements on performance and recovery [24]. Training loads and dietary intake were similar between placebo and creatine groups. The creatine group was given a recovery drink after training that included 20 g of creatine compared to placebo. There were similar body mass gains in both groups and no differences in recovery or performance markers. Further higher-powered studies are needed to better understand creatinine timing.

Muscle gain and loss prevention

The combination of resistance training with supplementation has become a major focus for research. Burke et al. performed a systematic review and meta-analysis of randomized controlled trials that combine creatine supplementation with resistance training [25]. They reported that creatine supplements enhance skeletal muscle hypertrophy when combined with resistance training. Small improvements of 0.10-0.16 cm in upper and lower body muscle thickness were shown. Young adults had a greater yet modest benefit in muscle hypertrophy (ES = 0.23 (95% CrI: 0.01, 0.44)) compared to older adults (ES = 0.06 (95% CrI: -0.10, 0.21)). Systematic reviews have shown possible improvement in upper and lower limb performances in short-term exercise [26]. Mielgo-Ayuso et al. performed a systematic review and meta-analysis on the effect of creatine on soccer player performance [27]. Creatine improved anaerobic performance, specifically on the Wingate test (standardized mean differences, 2.26; 95% CI, 1.40-3.11; p < 0.001), which measures muscle performance power and anaerobic capacity over 30 seconds. However, there were no improvements in aerobic performance (standardized mean differences, -0.05; 95% CI, -0.37-0.28; p = 0.78) as well as strength, single jump, single sprint, and agility (standardized mean difference, 0.21; 95% CI, -0.03-0.45; p = 0.08) [27].

Creatine has also been investigated as a beneficial supplement for elderly individuals. Sarcopenia is the loss of muscle mass and strength with aging with decreased physical performance, muscle quantity, and muscle quality [28]. This leads to loss of mobility, increased risk of falls, loss of bone density, higher risk of fracture, higher postoperative complications, longer hospitalization, and increased risk of mortality [29]. The prevalence is thought to be around 5%-13% in patients aged 60 years or older and 11%-50% in those aged 80 and older [30]. Older adults typically have reduced bone density, muscle mass, and, in some instances, cognitive function [31]. Less than 12% of adults older than 55 years of age exercise for more than 150 minutes per week. Furthermore, less than 20% perform resistance training two days or more a week. Some studies have reported no effect with use of supplemental creatine for lean muscle mass or performance [32,33]. However, many of these studies did not include resistance training in addition to creatine supplement.

Chami et al. performed a double-blind randomized trial to determine whether high-dose creatine dosed 0.3 g/kg/day (average of 25 g per day) has greater benefits for muscle strength, endurance, and function compared to moderate-dose creatine dosed 0.1 g/kg/day (average of 8 g per day) [34]. They found increases in leg press and chest press strength as well as endurance; however, this was independent of dose and was not significantly different from placebo. This study suffered from low sample size and lack of baseline dietary intake, which may have influenced results. Devries and Phillips performed a meta-analysis comparing creatine and resistance training versus resistance training alone [35]. Randomized controlled trials were included if they studied adults older than 45 years with a resistance training period greater than six weeks. Ten studies were included, with 357 subjects aged 55-71 years. Creatine and resistance training increased total body mass (p = 0.004), chest press (p = 0.004), and leg press (p = 0.02) one-rep max compared to resistance training alone. However, this meta-analysis and any subgroup analyses were limited by low number of included trials and heterogeneity with differences between trials in terms of sex and training methods. Chilibeck et al. performed a more recent systematic review and meta-analysis of randomized controlled trials examining creatine and resistance training in older adults [36]. Twenty-two studies were included, with 721 subjects aged 57-70 years who were randomized to creatine supplementation or placebo with resistance training two to three days per week for 7-52 weeks. Creatine and resistance training cohort showed increases in lean mass (p < 0.00001), leg press (p = 0.01), and chest press (p = 0.0002) [36]. This too was limited by heterogeneity as eight of these studies included a loading phase, while the remaining studies had various doses from 3 to 5 g per day.

In terms of adolescent and pediatric patients, there is limited evidence to support creatine supplementation [37]. A systematic review by Metzger et al. found that the majority of studies in this population were of poor quality and did not demonstrate consistent results for improvement in athletic performance [37].

Role in cognitive function

Investigators have sought to find a possible link between creatine and improved brain cognition. Given that brain cells also use creatine for energy, it is thought that creatine supplementation may meet increased neuronal ATP demands with cognition. Dechent et al. demonstrated an increase in the mean concentration of total creatine by 8.7% (0.6 mM, p < 0.001) across brain regions after a 20 g per day four-week regimen of creatine supplement [38]. However, the cognitive effects of creatine supplementation have been mixed in the literature. Rae et al. examined the effect of creatine supplements in young adult vegetarians in a double-blind randomized controlled trial [39]. Creatine supplementation had a positive effect on abstract reasoning (Raven advanced progressive matrices (RAPM)) and working memory (Weschler auditory backward digit span (BDS)) (p < 0.0001). Sandkühler et al. performed a similar double-blind randomized controlled trial with 123 individuals (half vegetarian and half omnivore) taking 5 g creatine per day for six weeks [31]. Tests for working memory (BDS, p = 0.067), abstract reasoning (RAPM, p = 0.315), and eight exploratory cognitive tests did not reach significance. Larger well-designed studies are needed to determine if there are cognitive benefits with creatine use.

Role in postoperative recovery

Malnutrition has been recognized to play a role in postoperative outcomes in orthopedic surgery. Patients undergo a catabolic inflammatory state postoperatively. This inflammatory state and, in some cases, immobilization can lead to postoperative muscle and strength losses [40,41]. Johnston et al. compared the effect of placebo versus creatine supplementation on muscle mass and strength after limb immobilization [42]. The creatine cohort showed better maintenance of upper-limb lean tissue mass (+0.9%) compared to placebo (-3.7%) during immobilization (p < 0.05). Elbow flexion strength and endurance were noted to decrease in both groups; however, the creatine cohort mitigated the reduction in flexion strength (creatine -4.1% versus placebo -21.5%, p < 0.05) and endurance (creatine -9.6% versus placebo -43%, p < 0.05). There was a similar effect on elbow extension strength (creatine -3.8% versus placebo -18.3%, p < 0.05) and endurance (creatine -6.5% versus placebo -35%, p < 0.05).

It is known that malnourished patients have increased risk for complications, longer lengths of stay, higher readmission rates, and higher mortality [43]. Protein and amino acid supplementation have been suggested to improve speed of recovery after orthopedic surgery [44]. However, the role for creatine in postoperative recovery has not yet been clearly established from the few studies to date.

Roy et al. conducted a randomized trial to evaluate whether creatine supplementation improved recovery after 37 total knee arthroplasties [45]. Patients received either placebo or creatine monohydrate (10 g/day for 10 days preoperatively, followed by 5 g/day for 30 days postoperatively). Creatine supplementation did not improve muscle strength or overall recovery. Another randomized controlled trial by Tyler et al. included 60 patients randomized to placebo or creatine supplementation following anterior cruciate ligament (ACL) reconstruction [46]. The authors reported no differences between the creatine and placebo groups in strength, power, single-leg hop test performance, or knee outcome score.

There has been growing interest in possible supportive aids for peripheral nerve regeneration. Helvacioglu et al. investigated the effect of creatine on rat sciatic nerve recovery following crush injury [47]. Given oxidative stress and inflammatory changes distal to nerve injury, the authors sought to determine if creatine was effective in supporting nerve regeneration with reduced apoptosis and oxidative stress. Light microscopic measurements of entire cross-sections of rat sciatic nerves showed significantly higher numbers of fibers in the trauma + creatine group compared to the trauma group (p < 0.001). There was also a significantly higher number of blood vessels in each nerve section in the trauma + creatine group compared to the trauma-only group (p < 0.001).

Other areas of interest include muscle injury and recovery. One study examined the myoprotective effects of creatine and whey protein using rat extensor digitorum longus [48]. The authors used bupivacaine to chemically induce muscle damage and followed the muscles for 14 days. Subjects with creatine supplementation showed significantly more intact fibers (19%, p = 0.002) at day seven as well as larger cross-sectional area of regenerating and intact fibers (p = 0.010).

While these animal studies may show promise, further clinical evidence is needed to elucidate the effect on human subjects. Overall, there appears to be limited risk with the use of creatine in postoperative recovery; however, studies thus far have not shown clear benefits for preoperative and postoperative supplementation.

## Conclusions

Orthopedic surgeons are likely to encounter patients who are taking or seeking information about nutritional supplements. Creatine may provide benefits in the general population and in the elderly when combined with resistance training. However, evidence supporting creatine supplementation in the adolescent and pediatric populations remains limited. Creatine use should be cautioned in patients at risk for kidney disease or those with poor kidney function. Thus far, studies have not shown a clear benefit of preoperative and postoperative creatine supplementation in patients undergoing orthopedic surgery.
